# Application of Dynamically Constrained Interpolation Methodology to the Surface Nitrogen Concentration in the Bohai Sea

**DOI:** 10.3390/ijerph16132400

**Published:** 2019-07-06

**Authors:** Quanxin Zheng, Xiaona Li, Xianqing Lv

**Affiliations:** 1 Key Laboratory of Physical Oceanography, Qingdao Collaborative Innovation Center of Marine Science and Technology (CIMST), Ocean University of China, Qingdao 266100, China; 2 Qingdao National Laboratory for Marine Science and Technology, Qingdao 266100, China

**Keywords:** surface nitrogen concentration (SNC), dynamically constrained interpolated methodology (DCIM), adjoint method

## Abstract

Observations of ocean pollutants are usually spatiotemporally dispersive, while it is of great importance to obtain continuous distribution of ocean pollutants in a certain area. In this paper, a dynamically constrained interpolated methodology (DCIM) is proposed to interpolate surface nitrogen concentration (SNC) in the Bohai Sea. The DCIM takes the pollutant transport advection diffusion equation as a dynamic constraint to interpolate SNCs and optimizes the interpolation results with adjoint method. Feasibility and validity of the DCIM are testified by ideal twin experiments. In ideal experiments, mean absolute gross errors between interpolated observations and final interpolated SNCs are all no more than 0.03 mg/L, demonstrating that the DCIM can provide convincing results. In practical experiment, SNCs are interpolated and the final interpolated surface nitrogen distribution is acquired. Correlation coefficient between interpolated and observed SNCs is 0.77. In addition, distribution of the final interpolated SNCs shows a good agreement with the observed ones.

## 1. Introduction

The Bohai Sea is the largest and the only semiclosed inland sea in China, surrounded by land on its three sides. It connects to the north of the Yellow Sea through the Bohai Strait. The weak water exchange causes a poor self-purification ability of the Bohai Sea, making it difficult to be restored in a short time if the marine ecosystem is severely damaged. According to statistics, the sewage water of over 40 rivers flows into the Bohai Sea and the volume is nearly 890 × 10^8^ m^3^ [1], which inevitably aggravates the environmental problems, such as ocean eutrophication (the accumulation of nutrients: nitrogen, phosphorus, etc.). Moreover, the severe deterioration in marine environment has badly affected the development of fishery and the Bohai Sea is gradually losing its function as a fishing ground [2]. To maintain sustainable development, relevant researches about marine pollutants have been conducted. The mathematical models are considered as the most direct and effective way for quantification [3], and with help of a mathematical model knowing more about the temporal and spatial distributions of pollutants in the Bohai Sea plays an important role in environment restoration.

Activities in coastal oceans can help to speed up economic construction, but meantime it will cause serious pollution to marine ecosystems. A number of numerical studies have been carried out to simulate pollutant dispersion [4,5,6,7]. A two-dimensional water quality model was developed and applied to analyze and optimize the ecological programs, and it can simulate key model variables (NH_4_^+^-N, PO_4_^3−^-P, chemical oxygen demand, and water level) [8]. Lee et al. [9] established an advection-dispersion model for pollutant transport simulation to analyze the influence of tidal currents on the concentration distribution. Gupta et al. [10] utilized numerical modeling to determine the sewage assimilative ability and found that the water quality was badly deteriorated due to the multiple sewage discharge. Periáñez [11] developed a particle-tracking model constituted by an off-line running hydrodynamic module to simulate the dispersion of pollutants. A three-dimensional numerical model of gravity flows was introduced in the research of Huang et al. [12], which was used to investigate the distribution features of various pollutants discharged at different positions in a wide river. Li et al. [13] simulated the temporal and spatial distribution of pollutants of the Bohai Sea in twin experiments with the adjoint assimilation method.

Interpolation methods, such as the Kriging, Cressman, spline, and polynomial interpolations, are widely used in the numerical model to obtain an integrated field based on sparse observations. Bargaoui and Chebbi [14] applied Kriging methods to evaluate the spatial and temporal variability of rainfall. Jeffrey et al. [15] adopted a thin plate smoothing spline and the ordinary Kriging to get daily climate variables and rainfall, respectively. A modified Cressman method was proposed in the study of Liu et al. [16], where the influence radius was modified to produce relatively accurate distributions. Wang et al. [17] applied the Cressman interpolation method to calculate the monthly mean distribution of total nitrogen to study the initial filed of pollution in the Bohai Sea. Guo et al. [18] introduced the surface spline interpolation to a two-dimensional tidal model and illustrated the feasibility and practicability of the method.

The adjoint assimilation method has been applied in oceanography for decades [19,20,21,22]. Zhang et al. [23] used the adjoint method in a two-dimensional tidal model to study the characteristics of bottom friction parameterizations. A three-dimensional cohesive sediment transport model with the adjoint assimilation method was established in Wang et al. [24] to get better simulation results of parameters. Furthermore, Mao et al. [25] developed the dynamically constrained interpolation methodology (DCIM), where the dynamic constraints were combined with the statistical information of observations to interpolate the suspended sediment concentrations. In this paper, DCIM will be applied to interpolate the surface nitrogen concentration in the Bohai Sea. The rest of the paper is organized as follows. Section 2 describes the dynamic constraint model, observation information, and details of the DCIM. Section 3 gives the results of numerical experiments. Section 4 concludes the whole work.

## 2. Materials and Methods

### 2.1. The Dynamical Model

Considering the convection and diffusion processes, the governing equation of marine pollutant transport model is presented as follows [17,26,27]

(1) $$\frac{\partial C}{\partial t}+u\frac{\partial C}{\partial x}+v\frac{\partial C}{\partial y}+w\frac{\partial C}{\partial z}=\frac{\partial }{\partial x}(A_{H}\frac{\partial C}{\partial x})+\frac{\partial }{\partial y}(A_{H}\frac{\partial C}{\partial y})+\frac{\partial }{\partial z}(K_{H}\frac{\partial C}{\partial z})-rC$$

where *C* denotes the concentration of pollutants; *t* and *x*, *y*, *z* are the symbols of time and space, respectively; *u* and *v* represent the horizontal velocities (in *x* and *y* directions, respectively) and *w* represents the vertical velocity (in *z* direction); *A_H_* and *K_H_* denote the horizontal and vertical diffusion coefficients (*A_H_* = 100 m^2^/s, *K_H_* = 0.00001 m^2^/s), respectively; *r* is the pollutant attenuation coefficient, and *r* = 0, which means that the pollutant is treated as conservative substance [17]. For the finite difference scheme readers can be referred to the Appendix A.

The open boundary of the model is set at 122.5° E, where a no-gradient condition and constant condition are used at the outflow boundary and the inflow boundary, respectively.

### 2.2. Observations and Model Setting

**Figure 1:**
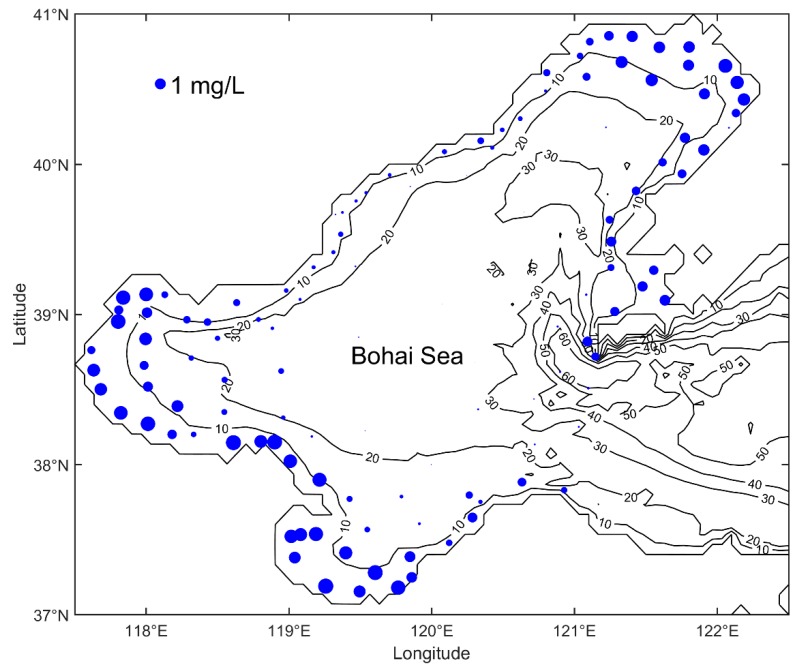
Topography of the Bohai Sea (depth in meters) and distribution of observation points in May 2009. The size of each point indicates surface nitrogen concentration.

The marine environmental monitoring data in the Bohai Sea and the north Yellow Sea, provided by the North China Sea Environmental Monitoring Center, State Oceanic Administration, includes the data of February, May, August and October of each year. Nitrate, phosphate, pH, etc. are monitored in order to investigate the spatiotemporal distribution of different pollutant elements and then diagnose marine pollution matter [16]. The distribution of observation points is shown in Figure 1 and the date of observations is given in Table 1.

The monitoring data in 2009 are analyzed in practical experiments in this paper. The computational domain is the Bohai Sea (37° N–41° N, 117.5° E–122.5° E) with a 4′ × 4′ grid resolution. The computing time is 30 days and the time step is set to be 6 h. The three-dimensional Regional Ocean Model System (ROMS) provides the hydrodynamic flow field used in numerical experiments of present study [27].

### 2.3. Dynamically Constrained Interpolation Methodology

According to the study of Yaremchuk and Sentchev [28], the dynamically unconstrained interpolation method and the dynamically constrained interpolation method (DCIM) are two parts of interpolation methods, and the DCIM is used in the model to obtain the interpolation of observations. In addition, the adjoint method is used to optimize the interpolation results.

#### 2.3.1. The Adjoint Methods

To optimize the interpolation results, the misfit between interpolation results and observations should be gradually reduced, which is described by the cost function and defined as [17]

(2) $$J=\frac{1}{2}{\sum K_{C}(C_{i,j,k}-{\bar{C}}_{i,j,k})}^{2}$$

where *C*_*i*,*j*,*k*_ and ${\bar{C}}_{i,j,k}$ denote the interpolation results and the observation data at the point (*i*,*j*,*k*), respectively; *K_C_* represents the weighting matrix whose element equals to 1 when the observations are available; otherwise, *K_C_* = 0.

The governing equation of marine pollutant transport model (1) can be written as

(3) $$F=\frac{\partial C}{\partial t}+u\frac{\partial C}{\partial x}+v\frac{\partial C}{\partial y}+w\frac{\partial C}{\partial z}-\frac{\partial }{\partial x}(A_{H}\frac{\partial C}{\partial x})-\frac{\partial }{\partial y}(A_{H}\frac{\partial C}{\partial y})-\frac{\partial }{\partial z}(K_{H}\frac{\partial C}{\partial z})+rC$$

Based on the Lagrange multiple method, the Lagrange function can be written as

(4) $$L=J+\int_{\Omega }(C*F)d\Omega$$

where C* represents the adjoint variable of *C*; Ω denotes the computational domain.

The adjoint model of the pollution transport model is calculated from Equation (5). The gradients of the cost function with respect to model parameters can be calculated by Equation (6):

(5) $$\frac{\partial L}{\partial C}=0$$

(6) $$\frac{\partial L}{\partial p}=0$$

where *p* stands for the model parameters.

In this paper, the adjoint equation and the gradient can be written as Equations (7) and (8), respectively.

(7) $$\begin{array}{l} -\frac{\partial C^{*}}{\partial t}-\frac{\partial }{\partial z}(K_{H}\frac{\partial C^{*}}{\partial z})\\ =\frac{\partial uC^{*}}{\partial x}+\frac{\partial vC^{*}}{\partial y}+\frac{\partial wC^{*}}{\partial z}+\frac{\partial }{\partial x}(A_{H}\frac{\partial C^{*}}{\partial x})+\frac{\partial }{\partial y}(A_{H}\frac{\partial C^{*}}{\partial y})-K_{C}(C-\bar{C}) \end{array}$$

(8) $$\begin{array}{cc} \frac{\partial J}{\partial C^{1}} & ={\left(\frac{\partial C^{*}}{\partial t}\right)}^{1}+{\left(\frac{\partial uC^{*}}{\partial x}\right)}^{1}+{\left(\frac{\partial vC^{*}}{\partial y}\right)}^{1}+{\left(\frac{\partial wC^{*}}{\partial z}\right)}^{1}\\ & +\frac{\partial }{\partial x}{\left(A_{H}\frac{\partial C^{*}}{\partial x}\right)}^{1}+\frac{\partial }{\partial y}{\left(A_{H}\frac{\partial C^{*}}{\partial y}\right)}^{1}+\frac{\partial }{\partial z}{\left(K_{H}\frac{\partial C^{*}}{\partial z}\right)}^{1} \end{array}$$

where the superscript 1 denotes the SNC at the first iteration step.

#### 2.3.2. The Process of DCIM

The DCIM contains the following steps, described as [25] follows.

Step 1. Propose a guess value of the parameters in the model.

Step 2. Acquire the interpolation of observations through forward model.

Step 3. Calculate the cost function and obtain the Lagrange multiple through adjoint model.

Step 4. Based on Equation (6), acquire the gradients of the cost function with respect to the parameters of the model and adjust the parameters along the opposite direction of the gradient.

Step 5. Stop calculating when the preset ending condition is satisfied; otherwise, go to step 2 and continue iterating.

## 3. Numerical Results

### 3.1. Verification of the DCIM

In this part, we testified the feasibility and validity of the DCIM by ideal experiments. The observations used in ideal experiments were generated by integrating the given distribution of nitrogen over time. In order to maintain the universality, the initial guess values were set to be half of the max value of nitrogen concentration. Similar conclusions were drawn when other initial guess values were taken. Statistic results of other initial guess values are given in Appendix B.

#### 3.1.1. Application of the DCIM in Ideal Twin Experiments

As mentioned by Elbern et al. [29], the validity of the assimilated or interpolated results can only be testified by the observations that were not assimilated or interpolated. Therefore, one-fifth of the total observations were randomly selected as observations that were not interpolated but only used for verification, and these observations were named as checking observations. The other observations were named as interpolated observations, which were to be interpolated with the DCIM. By this cross-validation, it can be distinguished that whether the interpolated observations were overfitted or not. If the interpolated observations were overfitted, there would be large misfit between simulation results and checking observations [30].

To eliminate the contingency induced by selection of checking observations, all idealized observations were randomly divided into five subsets and every subset was taken as the checking observations by turns. Therefore, there were five twin experiments, which were named as IE_11–IE_15, respectively. The statistics of these twin experiments are listed in Table 2. The Cressman interpolation method [31] was introduced to the ideal twin experiments IE_11-IE_15 so that the quality of results can be assessed. The comparison is presented in Appendix C. In order to quantify the difference between interpolated SNCs and observations, mean absolute gross error (MAGE) and mean normalized gross error (MNGE) were calculated as follows

(9) $$\mathrm{MAGE}=\frac{1}{N}\sum_{i=1}^{N}\left|I_{i}-O_{i}\right|$$

(10) $$\mathrm{MNGE}=\frac{1}{N}\sum_{i=1}^{N}\left[\left(\left|I_{i}-O_{i}\right|\right)/O_{i}\right]$$

where *N* is the number of observations and *I* and *O* are the interpolated SNCs and observations, respectively.

**Figure 2:**
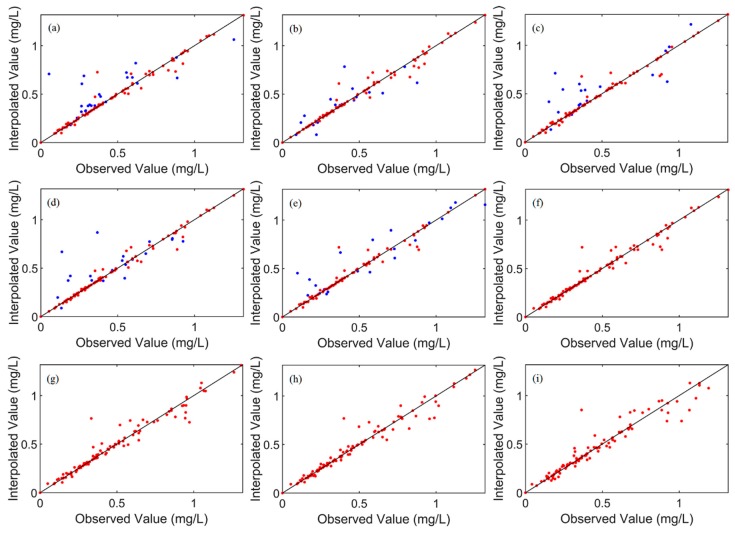
Comparison of simulated and observed surface nitrogen concentrations (SNCs), including interpolated observations (red dots) and checked observations (blue dots), for (**a**) IE_11, (**b**) IE_12, (**c**) IE_13, (**d**) IE_14, (**e**) IE_15, (**f**) IE_21, (**g**) IE_31, (**h**) IE_32, and (**i**) IE_33. The 1:1 lines are shown for reference in all the panels.

In the five twin experiments, the rate of decline in MAGEs between checking observations and corresponding interpolated SNCs (K3) were 66.7%, 70.8%, 63.6%, 57.1%, and 71.4%, respectively; and the errors were no more than 0.12 mg/L. What is more, the MNGEs between checking observations and interpolated SNCs (K4) were all reduced by at least 50%. Besides, at the first iteration step, the MNGEs between interpolated observations and corresponding interpolated SNCs (K2) were all larger than 120%, while after applying the DCIM, K2 were all less than 6%. Thus, it can be demonstrated that the interpolated observations were not overfitted. Figure 2 shows that most dots were near the 1:1 line, no matter whether the dot stands for interpolated observations or checking observations, which indicates that the DCIM was an effective tool to interpolate observations.

To make the fullest use of all observations, another twin experiment IE_21 was conducted. In IE_21 all observations were used to interpolate and the final MAGE was 0.02 mg/L, which was reduced by 92.9%, while the final MNGE was 6.06% (see Table 2). Comparison between interpolated SNCs and prescribed observations is shown in Figure 2f. The correlation coefficient was near 1.00 on the whole, meaning that the final interpolated results were almost equal to the artificial observations. Thus, we can say that the DCIM was a feasible and effective method to interpolate the SNCs.

#### 3.1.2. Sensitivity to Observational Errors

In the real ocean environments, the observations can be contaminated by noises. Therefore, another three twin experiments, named by IE_31, IE_32, and IE_33, respectively were conducted, in which random perturbations were added to the prescribed observations. The maximum percentages of observation errors were 10%, 20%, and 30% in three experiments, respectively. The comparison between interpolated SNCs and observations were shown in Figure 2g–i, and the results indicated that the final interpolated SNCs were close to the observations in all three twin experiments. Moreover, the statistics of MAGEs and MNGEs shown in Table 2 also demonstrated that although the observations contained noises, the DCIM can still perform well when used to interpolate the SNCs. This means that the interpolation results may still be convincing when the DCIM was adopted in the practical situation.

### 3.2. Practical Applications

**Figure 3:**
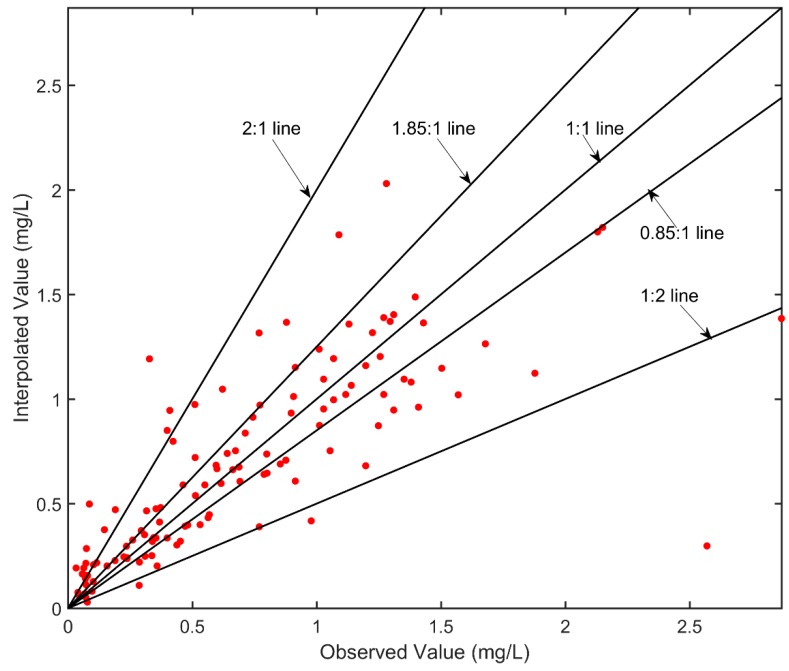
Comparison of interpolated and observed SNCs. The 2:1, 1.85:1, 1:1, 0.85:1, and 1:2 lines are shown for reference.

In this section, the observed data of SNCs were used to carry out practical experiments. The final MAGE and MNGE were 0.21 mg/L and 47.9%, respectively (Table 3), which were both reduced by more than 55%. The mean value and the standard deviation of the observed SNCs were 0.69 and 0.55 mg/L, respectively, while those of the interpolated SNCs were 0.69 and 0.46 mg/L, respectively. The results indicated that the interpolated SNCs were almost equal to the observed SNCs. Figure 3 showed the scatterplot to compare interpolated SNCs and observed SNCs visually. The 2:1, 1.25:1, 1:1, 0.85:1, and 1:2 lines were shown for reference. For 84.3% of the observations, the ratio of interpolated SNCs to the observed was between 0.5 and 2; for 11.6%, the ratio was over 2 and for 4.1%, the ratio was below 0.5. It was obvious that the closer the ratio was to 1, the close the interpolated SNCs were to the observed SNCs. For 53.7% of the observations, the ratio was between 0.85 and 1.25. What is more, the correlation coefficient between the interpolated SNCs and the observed SNCs was 0.77.

**Figure 4:**
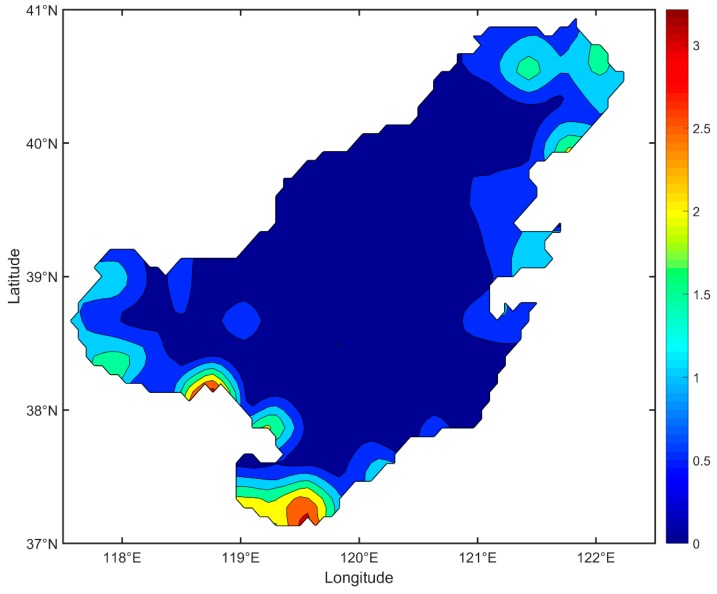
The final distribution of the interpolated surface nitrogen concentration (unit: mg/L).

**Figure 5:**
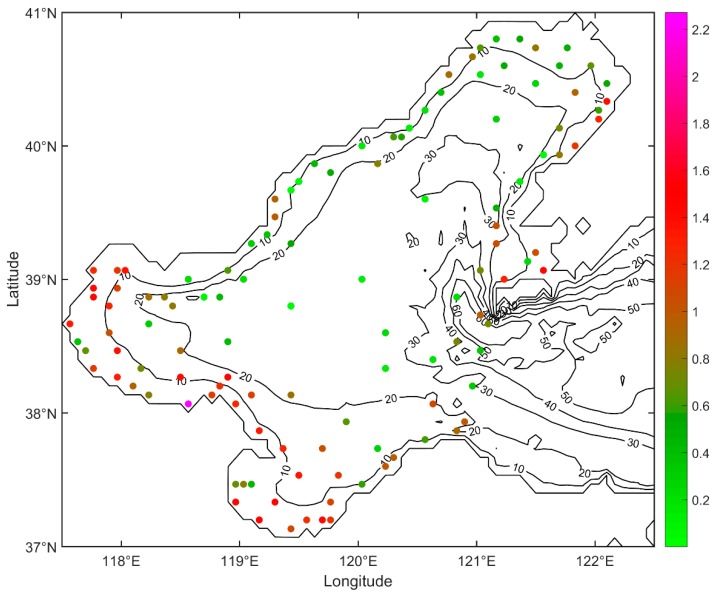
Mean absolute gross error (MAGE) between each interpolated observation and interpolated SNC (unit: mg/L).

**Figure 6:**
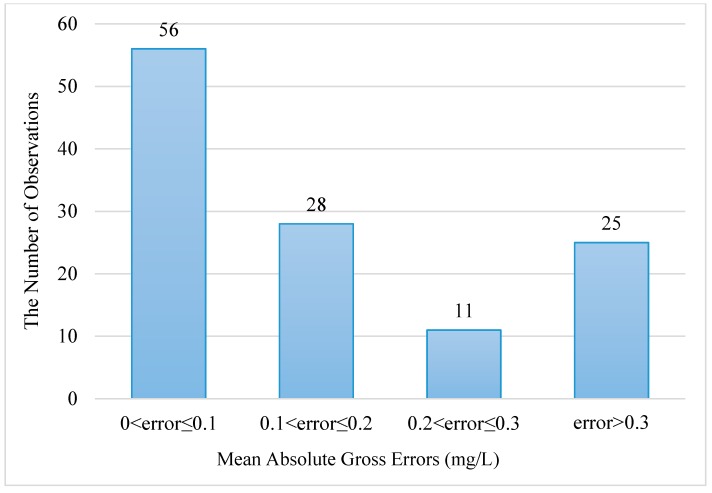
Statistics of MAGEs.

The statistical results mentioned above indicated that the interpolated SNCs with DCIM were coherent with the observed SNCs. The final distribution of the interpolated surface nitrogen concentration was given in Figure 4. The MAGE between each interpolated observation and interpolated SNC was shown in Figure 5. Statistics of MAGEs was shown in Figure 6. By statistics, we can know that 46.3% (56/121) of the MAGEs were no more than 0.1 mg/L and only 20.7% (25/121) of the MAGEs were over 0.3 mg/L. Figure 4 showed that high concentration appears in the three bays, while in the central Bohai Sea the concentration was low, and comparing with Figure 1 it showed a good agreement with the observed nitrogen concentration distribution.

## 4. Conclusions

In this paper, we interpolated the surface nitrogen concentration with the dynamically constrained interpolation methodology (DCIM). The pollutant transport model was taken as dynamic constraint and the interpolated results were optimized iteratively with the adjoint method.

The feasibility and validity of DCIM were testified with prescribed observations in ideal twin experiments. The statistics and the scatterplot of twin experiments illustrated that the interpolated SNCs with DCIM were close to the prescribed observations and that the interpolated results were still convincing when noises were added to the prescribed observations. In practical experiment, the observed data were used to interpolate the surface nitrogen concentration with DCIM. The correlation coefficient between interpolated SNCs and observed SNCs was 0.77. The distribution of final interpolated surface nitrogen concentration shows a good agreement with the observations. The interpolated results in ideal experiment and in practical experiment demonstrated that the DCIM can be an effective method to interpolate the spatial and temporal distributing observations.

## Figures and Tables

**Table 1:** Observation information: location and date.

Longitude (°N)	Latitude (°E)	Date	Longitude (°N)	Latitude (°E)	Date
119.7051	39.928	2	119.7881	37.7864	13
119.375	39.6778	5	121.5944	40.7764	13
120.9278	37.8292	6	119.5333	38.225	14
120.6319	37.8819	6	119.1611	38.1861	14
119.4708	39.7542	7	120.3417	37.75	14
120.4236	40.1083	7	119.5486	37.5667	14
120.0889	40.0833	7	119.425	37.7708	14
118.3333	38.2	7	120.2639	37.7958	14
120.3444	40.1542	7	120.2861	37.6458	14
118.1298	39.1307	7	121.3306	40.6792	14
118.6347	39.0768	8	121.5417	40.5597	14
120.8069	40.6083	8	118	39.1333	14
121.0847	40.5819	8	117.8389	39.1111	14
118.7874	38.9652	9	118.9597	38.3111	15
120.4944	40.2278	10	118.9453	38.6217	15
120.6208	40.3028	10	118.5483	38.3489	15
122.1403	40.5444	10	118.55	38.5625	15
122.0569	40.6542	10	117.8083	39.0292	15
120.7972	40.4861	11	118.0069	39.0111	15
119.3625	39.5333	11	117.8042	38.9514	15
118.2861	38.964	11	122.0819	40.2417	16
118.4303	38.9472	11	118.5	38.8403	16
121.7986	40.6583	11	118.3167	38.7083	16
121.8042	40.7792	11	117.6167	38.7625	16
121.0403	40.7194	12	121.775	40.175	16
121.1083	40.8139	12	118.8028	38.1528	18
121.2417	40.8528	12	118.9008	38.1483	18
121.4056	40.85	12	122.1319	40.3389	19
119.9969	37.9994	13	121.9111	40.4681	19
120.7222	38.1333	13	122.1875	40.4292	19
120.325	38.3667	13	118.0139	38.5181	20
119.9139	37.6056	13	119.4889	38.8472	21
119.0792	39.0986	21	121.1472	38.7181	24
119.5403	39.8089	21	121.2833	39.0181	24
118.8833	38.9069	21	121.0917	38.8181	24
121.6167	40.0111	21	119.2583	37.1903	24
121.7528	39.9347	21	121.2208	40.2445	25
118.1833	38.2	21	120.0722	39.0667	26
121.9069	40.0944	21	120.8833	38.9194	26
118.2194	38.3889	21	121.082	39.1305	26
119.4956	37.1531	21	117.9861	38.6597	26
117.6833	38.5014	21	117.9972	38.8361	26
119.3978	37.4111	21	117.6333	38.6278	26
117.8222	38.3444	21	118.6117	38.1444	26
118.0139	38.2708	21	119.0403	37.3792	27
119.6042	37.2806	21	119.0825	37.5319	27
119.3264	39.6639	22	119.0167	37.5208	27
119.4667	39.318	22	119.2153	37.8986	27
121.2458	39.6292	22	119.1889	37.5361	27
121.4319	39.8208	22	120.9667	37.9917	28
121.2583	39.4833	22	120.3028	38.6792	28
119.1726	39.3123	23	120.7167	38.4361	28
118.9805	39.1583	23	121.0306	38.25	28
121.2556	39.3111	23	120.9	38.6167	28
121.5556	39.2944	23	121.0972	38.5083	28
121.4778	39.1847	23	119.0111	38.0222	28
121.6333	39.0931	23	120.1222	37.4778	30
120.6458	39.682	24	119.8611	37.2486	30
120.2458	39.925	24	119.8481	37.3861	30
119.85	39.85	24	119.7667	37.1792	30
119.3125	39.4139	24			

**Table 2:** Statistics of the ideal experiments.

	K1		K2		K3		K4	
Expt	Initial	Final	Initial (%)	Final (%)	Initial	Final	Initial (%)	Final (%)
IE_11	0.26	0.01	124.96	5.29	0.33	0.11	157.20	77.67
IE_12	0.28	0.01	135.45	4.00	0.24	0.07	118.44	28.00
IE_13	0.27	0.01	134.51	4.91	0.33	0.12	123.47	47.18
IE_14	0.30	0.01	131.71	3.74	0.21	0.09	135.63	50.93
IE_15	0.27	0.01	135.35	5.13	0.28	0.08	121.89	37.02
IE_21	0.28	0.02	132.47	6.06	—	—	—	—
IE_31	0.28	0.02	134.46	8.20	—	—	—	—
IE_32	0.28	0.02	134.27	7.68	—	—	—	—
IE_33	0.29	0.03	140.18	10.62	—	—	—	—

K1 is MAGEs between the interpolated observations and the interpolated SNCs (mg/L); K2 is MNGEs between the interpolated observations and the interpolated SNCs; K3 is MAGEs between the checking observations and the interpolated SNCs (mg/L); K4 is MNGEs between the checking observations and the interpolated SNCs.

**Table 3:** Statistics of practical experiment.

K1		K2	
Initial	Final	Initial (%)	Final (%)
0.48	0.21	120.74	47.90

K1 is MAGEs between the interpolated observations and the interpolated SNCs (mg/L); K2 is MNGEs between the interpolated observations and the interpolated SNCs.

## Appendix A. Difference Form of Equation (1) and Equation (5)

For Equation (1), the difference form was as follows

(A1) $$\begin{array}{l} \frac{C_{i,j,k}^{l+1}-C_{i,j,k}^{l}}{\Delta t}-\left[\frac{K_{H}\left(C_{i,j,k+1}^{l+1}-C_{i,j,k}^{l+1}\right)}{\Delta z_{k+1/2}\cdot \Delta z_{k+1}}-\frac{K_{H}\left(C_{i,j,k}^{l+1}-C_{i,j,k-1}^{l+1}\right)}{\Delta z_{k+1/2}\cdot \Delta z_{k}}\right]=\\ \frac{u_{i,j,k}^{l}\left(C_{i+1,j,k}^{l}-C_{i-1,j,k}^{l}\right)}{2\Delta x_{j}}-\frac{v_{i,j,k}^{l}\left(C_{i,j+1,k}^{l}-C_{i,j-1,k}^{l}\right)}{2\Delta y}-\frac{w_{i,j,k}^{l}\left(C_{i,j,k+1}^{l}-C_{i,j,k-1}^{l}\right)}{2\Delta z_{k}}+\\ \left[\frac{A_{H}\left(C_{i+1,j,k}^{l}-C_{i,j,k}^{l}\right)}{\Delta x_{j+1/2}\cdot \Delta x_{j+1}}-\frac{A_{H}\left(C_{i,j,k}^{l}-C_{i-1,j,k}^{l}\right)}{\Delta x_{j+1/2}\cdot \Delta x_{j}}\right]+\\ \left[\frac{A_{H}\left(C_{i,j+1,k}^{l}-C_{i,j,k}^{l}\right)}{{\left(\Delta y\right)}^{2}}-\frac{A_{H}\left(C_{i,j,k}^{l}-C_{i,j-1,k}^{l}\right)}{{\left(\Delta y\right)}^{2}}\right] \end{array}$$

For Equation (5), the difference form can be described as

(A2) $$\begin{array}{l} \frac{C_{i,j,k}^{*l-1}-C_{i,j,k}^{*l}}{\Delta t}-\left[\frac{K_{H}\left(C_{i,j,k+1}^{*l-1}-C_{i,j,k}^{*l-1}\right)}{\Delta z_{k+1/2}\cdot \Delta z_{k+1}}-\frac{K_{H}\left(C_{i,j,k}^{*l-1}-C_{i,j,k-1}^{*l-1}\right)}{\Delta z_{k+1/2}\cdot \Delta z_{k}}\right]=\\ \frac{\left(u_{i+1,j,k}^{l}C_{i+1,j,k}^{*l}-u_{i-1,j,k}^{l}C_{i-1,j,k}^{\}*l}\right)}{2\Delta x_{j}}+\frac{\left(v_{i,j+1,k}^{l}C_{i,j+1,k}^{*l}-v_{i,j-1,k}^{l}C_{i,j-1,k}^{*l}\right)}{2\Delta y}+\\ \frac{\left(w_{i,j,k+1}^{l}C_{i,j,k+1}^{*l}-w_{i,j,k-1}^{l}C_{i,j,k-1}^{*l}\right)}{2\Delta z_{k}}+\\ \left[\frac{A_{H}\left(C_{i+1,j,k}^{*l}-C_{i,j,k}^{*l}\right)}{\Delta x_{j+1/2}\cdot \Delta x_{j+1}}-\frac{A_{H}\left(C_{i,j,k}^{*l}-C_{i-1,j,k}^{*l}\right)}{\Delta x_{j+1/2}\cdot \Delta x_{j}}\right]+\\ \left[\frac{A_{H}\left(C_{i,j+1,k}^{*l}-C_{i,j,k}^{*l}\right)}{{\left(\Delta y\right)}^{2}}-\frac{A_{H}\left(C_{i,j,k}^{*l}-C_{i,j-1,k}^{*l}\right)}{{\left(\Delta y\right)}^{2}}\right]-\\ K_{C}\left(C_{i,j,k}^{l}-\bar{C_{i,j,k}^{l}}\right) \end{array}$$

## Appendix B. Statistic Results of Other Initial Guess Values

During calculation, there are three kinds of initial guess values, including the minimum, half and maximum of the nitrogen concentration. The simulation results were given in Table A1.

**Table A1:** Simulation results of different initial guess values.

Initial Guess	K1		K2	
	Initial	Final	Initial (%)	Final (%)
Minimum	0.37	0.02	85.39	5.48
Half	0.28	0.02	132.47	6.06
Maximum	0.90	0.02	344.03	8.58

K1 is MAGEs between the interpolated observations and the interpolated SNCs (mg/L); K2 is MNGEs between the interpolated observations and the interpolated SNCs (mg/L).

From Table A1 we know that after iterative optimization the final MAGE of the three initial guess values were all 0.02 mg/L. It meant that no matter what the initial guess value was, the final simulation results were almost the same. The reason why the final percentages of K2 were different was that due to the different initial guess values, the initial errors were different. So in the paper, half of the max value of nitrogen concentration was taken as the initial guess value.

## Appendix C. Comparison between the DCIM and Cressman Interpolation Method

In order to access the quality of results, the Cressman interpolation (CI) method was introduced to the ideal twin experiments IE_11-IE_15. Comparison of MAGEs between CI and DCIM was shown in Table A2.

**Table A2:** Comparison of MAGEs between the two methods (unit: mg/L).

	IE_11	IE_12	IE_13	IE_14	IE_15
DCIM	0.11	0.07	0.12	0.09	0.08
CI	0.23	0.22	0.29	0.28	0.35

In experiments, from IE_11 to IE_15, the MAGEs were reduced by 52.2%, 68.1%, 58.6%, 67.9%, and 77.1%, respectively, and errors were reduced by almost an order of magnitude. Through comparison, we can draw the conclusion that the DCIM was a much more effective interpolation method than the CI.
